# German Version of the Telehealth Usability Questionnaire and Derived Short Questionnaires for Usability and Perceived Usefulness in Health Care Assessment in Telehealth and Digital Therapeutics: Instrument Validation Study

**DOI:** 10.2196/57771

**Published:** 2024-11-21

**Authors:** Jannik Zimmermann, Harriet Morf, Florian Scharf, Johannes Knitza, Heidi Moeller, Felix Muehlensiepen, Michaela Nathrath, Till Orlemann, Thomas Voelker, Merlin Deckers

**Affiliations:** 1 Department of Psychology – Theory and Methodology of Counseling University of Kassel Kassel Germany; 2 Department of Internal Medicine 3, Rheumatology and Immunology University Hospital Erlangen, Friedrich-Alexander University (FAU), Erlangen-Nuremberg Erlangen Germany; 3 Deutsches Zentrum Immuntherapie (DZI) University Hospital Erlangen, Friedrich-Alexander University (FAU), Erlangen-Nuremberg Erlangen Germany; 4 Department of Psychology – Psychological Research Methods University of Kassel Kassel Germany; 5 Institute for Digital Medicine University Hospital of Giessen and Marburg, Philipps University Marburg Marburg Germany; 6 AGEIS Université Grenoble Alpes Grenoble France; 7 Center for Health Services Research, Faculty of Health Sciences Brandenburg Medical School Theodor Fontane Ruedersdorf bei Berlin Germany; 8 Palliative Care Team for Children Kassel Kassel Germany; 9 Department of Internal Medicine 1 University Hospital Erlangen, Friedrich-Alexander University (FAU), Erlangen-Nuremberg Erlangen Germany

**Keywords:** mHealth, mobile health, telehealth, usability, questionnaire validation, technology acceptance model, validity, questionnaire translation, Net Promoter Scale, NPS, usefulness, autoimmune chronic diseases, questionnaire, German, digital therapeutics, therapeutics, feasibility

## Abstract

**Background:**

The exponential growth of telehealth is revolutionizing health care delivery, but its evaluation has not matched the pace of its uptake. Various forms of assessment, from single-item to more extensive questionnaires, have been used to assess telehealth and digital therapeutics and their usability. The most frequently used questionnaire is the “Telehealth Usability Questionnaire” (TUQ). The use of the TUQ is limited by its restricted availability in languages other than English and its feasibility.

**Objective:**

The aims of this study were to create a translated German TUQ version and to derive a short questionnaire for patients—“Telehealth Usability and Perceived Usefulness Short Questionnaire for patients” (TUUSQ).

**Methods:**

As a first step, the original 21-item TUQ was forward and back-translated twice. In the second step, 13 TUQ items were selected for their suitability for the general evaluation of telehealth on the basis of expert opinion. These 13 items were surveyed between July 2022 and September 2023 in 4 studies with patients and family members of palliative care, as well as patients with chronic autoimmune diseases, evaluating 13 health care apps, including digital therapeutics and a telehealth system (n1=128, n2=220, n3=30, and n4=12). Psychometric exploratory factor analysis was conducted.

**Results:**

The analysis revealed that a parsimonious factor structure with 2 factors (“perceived usefulness in health care” and “usability”) is sufficient to describe the patient’s perception. Consequently, the questionnaire could be shortened to 6 items without compromising its informativeness.

**Conclusions:**

We provide a linguistically precise German version of the TUQ for assessing the usability and perceived usefulness of telehealth. Beyond that, we supply a highly feasible shortened version that is versatile for general use in telehealth, mobile health, and digital therapeutics, which distinguishes between the 2 factors “perceived usefulness in health care” and “usability” in patients.

**Trial Registration:**

German Clinical Trials Register DRKS00030546; https://drks.de/search/de/trial/DRKS00030546

## Introduction

### Telehealth, Mobile Health, and Digital Therapeutics

Telehealth is an umbrella term defined as “the provision of healthcare remotely by means of telecommunications technology,” whereas mobile health (mHealth) is an overlapping definition for “the use of mobile devices so that patients can solicit services electronically, use apps to verify information, and manage or monitor treatment or problems or other health-related issues” [1,2].

The exponential growth of telehealth and mHealth is revolutionizing health care delivery, because they have the potential to remove geographical barriers, increase access to medical services, and improve overall care quality [3]. Particularly, during the COVID-19 pandemic, there was a significant increase in the use of telehealth and mHealth, but the evaluation and its methodology have not matched the pace of its uptake [4,5]. Patients can thus receive support throughout the entire patient pathway, including app-supported diagnoses, therapy, and monitoring. Approved digital therapeutics (German: Digitale Gesundheitsanwendungen) are apps to improve treatment; their costs are covered by the statutory health insurance system in Germany. In the following discussion, we use “telehealth” to encompass the terms “mHealth” and “digital therapeutics.”

To achieve the greatest possible benefit from telehealth, usability is the key factor, especially with patients who have a cognitive limitation, are incapacitated by their disease, or are children [6-8]. This means that even evidence-based technology is not particularly effective for patient outcomes if it is difficult to use. This could be due to the technology itself or varying levels of eHealth literacy among users. According to Norman and Skinner [9], eHealth literacy is “the ability to seek, find, understand, and appraise health information from electronic sources and apply the knowledge gained to addressing or solving a health problem.”

Furthermore, measuring usability also protects patients from errors or experiencing harm. For example, if a certain telehealth system saves or displays medical data incorrectly and this leads to incorrect treatment, this inadequate usability can also disadvantage patients [10].

### Definition of Usability

The International Organization for Standardization (ISO) norm 9241-11 defines usability as [11] “the extent to which a system, product or service can be used by specified users to achieve specific goals with effectiveness, efficiency and satisfaction in a specified context of use.”

Yet, many researchers have used additional attributes to assess usability [12]. A systematic literature study by Weichbroth showed that in descending order of priority, learnability, memorability, cognitive load, errors, simplicity, and ease of use were additional attributes used to assess usability in mobile settings [13]. However, this literature study excluded publications from medical and health subject areas. Sousa et al [14] conducted a systematic review of usability questionnaires for eHealth and showed that many existing usability questionnaires share these attributes but generally lacked effectiveness, cognitive load, simplicity, and ease of use. Interestingly, Sousa and Lopez [14] reported that the majority of usability questionnaires not only assessed usability but also the perceived usefulness in health care. The questionnaires included questions aiming to assess whether telehealth was helpful in fulfilling health care needs, which is not part of the usability definitions listed. The usability attributes aim only to assess the app’s efficiency, for example, “duration spent on each screen” or the app’s effectiveness, for example, “number of steps required to complete a task” [13].

### Usability Questionnaires

On the one hand, authors of various studies used the single-item “Net Promoter Scale” (NPS) and the derived Net Promoter Score to evaluate telehealth [15-18]. However, the psychometric correlates of the Net Promoter Score are not clear, and it is also thought to measure satisfaction and acceptance [15]. We believe that the NPS seems to be a valuable instrument because it offers a straightforward, quantifiable, and very short measure of the user experience and is easy for patients to understand and respond to accurately. Its numerical scale facilitates clear aggregation and analysis of data, allowing for effective comparison over time and across patient groups. The categorization into promoters, passives, and detractors appears to provide actionable insights for further improvements and its widespread use across industries, including health care [19].

On the other hand, up to 38-item questionnaires were used to measure usability [20]. We have, therefore, decided to include the NPS in our study to assess its association with known usability attributes.

A closer look at usability questionnaires reveals a need for development. First, different questionnaires exist side by side, sometimes measuring only different facets of usability or usability-related constructs [14]. Second, many questionnaires have little empirical evidence regarding their psychometric properties or, third, they can only assess the usability of a specific or single technology [14]. Fourth, most of the questionnaires are only available in English. Accordingly, there are hardly any validated and appropriate questionnaires for usability studies in the German language [21]. As far as we know, only the German translation of the System Usability Scale—the origin of all usability questionnaires—seems to be available for wider use so far [22]. However, none of the 4 existing German versions is convincing [23]. The first 3 points also become clear when you look at the small number of questionnaires available in German.

Some questionnaires are available in German capture usability-related constructs, but do not focus directly on usability (eg, Mobile App Rating Scale-German [MARS-G] [24] and the User Experience Questionnaire [25]). The AttrakDiff questionnaire measures usability as merely 1 dimension among several others [26]. More specifically for the telehealth area, Altmann et al [27] published a German version and a short version of the “Telemedicine Perception Questionnaire” (TMPQ) in 2022. The original questionnaire includes 17 items designed to evaluate the patients’ impressions of home telecare, as well as to assess its potential risks and benefits. Thus, the TMPQ does not measure usability per se (see first point) and is limited to evaluating older patients receiving video consultations from a nurse (see third point). Moreover, the validation of the questionnaire is limited to only 32 and 10 participants in its validation study [28] (see second point). The German translation could be shortened after subgroup analysis to a short version with 5 items. The German version of the TMPQ showed sufficient reliability (Cronbach α=0.76) in Altmann’s study with 32 participants compared to the original study (Cronbach α=0.8). For the brief version, reliability was still acceptable with Cronbach α of 0.72.

There is another small number of questionnaires available in German whose area of application is very limited (eg, ISONORM 9241/110 on desktop apps [29]). Specific to the telehealth area, different authors in this field offered German translations of the mHealth App Usability Questionnaire (MAUQ) [30-32] (see third point). Moorthy et al [30] validated their translated MAUQ in a specific sample of 133 patients with cancer but, presumably due to the small sample size, the factor structure of the translated questionnaire was not further investigated. Kopka et al [31] provided a German version and a German short version in a sample of 148 patients using a symptom checker app in an emergency department in a randomized controlled trial. They showed that the original factor structure did not fit the data well, but no further investigation of the factor structure was conducted. In their validation study (n=53; see second point), Tacke et al [32] showed a strong positive correlation between their MAUQ translation and the System Usability Scale (SUS). However, the factor structure was not examined due to the sample size.

Considering these shortcomings, we still see a need for an appropriate questionnaire available in German. Our aim is to ensure that this German questionnaire is suitable for assessing the general usability of both telehealth and video consultations. In addition, the factor structure is to be evaluated on the basis of a sufficiently large dataset.

### Telehealth Usability Questionnaire

The Telehealth Usability Questionnaire (TUQ) by Parmanto et al [33] in 2016 measures all usability attributes except memorability, allows the evaluation of video consultations, and is the most used usability questionnaire [34,35]. The TUQ uses preexisting items from other questionnaires and is freely available following the Creative Commons license 4.0. The TUQ is recommended by other authors and by frameworks for assessing telehealth [14,17,33-35]. We, therefore, translated and validated the TUQ in German (see Methods for further information on the TUQ).

Bibiloni et al [36] published an exploratory factor analysis (EFA; 150 questionnaires) of the TUQ [33] relating to video consultation. They found that 2 factors were sufficient to model the observed data. After the questionnaire was adapted to 12 items by an expert team, a confirmatory factor analysis (269 questionnaires) was performed. Both factors could be measured with good reliability, but they were highly positively correlated. Despite adapting the items and shortening the questionnaire to 12 items, the main problem with the questionnaire was that no good differentiation between usability and perceived usefulness in health care could be achieved. Although the high factor correlation raised the question of whether respondents differentiate between these 2 aspects, a 1-factor model showed a clearly worse fit than the 2-factor model. A limit to the application of the short questionnaire of Bibiloni et al [36] was that the inclusion criteria merely required a single instance of a video consultation and thus did not allow for the general evaluation of apps in telehealth. Besides, as the factor analysis of Bibiloni et al [36] confirmed, the TUQ measures usability, as well as perceived usefulness in health care, and the name “Telehealth Usability and Usefulness Questionnaire” would be more appropriate.

The primary objective of this study was to develop and validate a German language version of the TUQ and compare it to the NPS. This adaptation aims to make the TUQ readily accessible and broadly applicable for evaluating telehealth usability within German-speaking populations. The second aim was to reduce the number of items in order to optimize the feasibility for use in general field studies in telehealth.

## Methods

### Stages of the Study

Stage I (April 2022-January 2023) consists of translation, adaptation, and pilot-testing of face validity as a method of construct validity. Stage II (July 2022-September 2023) consists of the development of a short-scale—psychometric testing and final item selection. The reporting of this study has been structured according to the recommendations of Streiner and Kottner [37] for reporting the results of studies of instrument and scale development and testing.

### Stage I: Translation, Adaptation, and Pilot User Testing

High-quality translations can only be produced if measurement instruments are linguistically replicated and culturally adapted as rigorously as possible. Sousa and Rojjanasrirat [38] presented a guideline on “Translation, adaptation and validation of instruments or scales for use in cross-cultural health care research.” In agreement with the lead author, Bambang Parmanto, we (MD and JZ) conducted translation and cross-cultural adaptation with a multidisciplinary expert committee. The members of the expert committee were selected on the basis of their knowledge and experience in the area of application of the questionnaire and their language skills, as reflected in their previous research activities and clinical experience. Translations were carried out by native speakers with proven expertise in the field of application and a high level of language skills in the target language of the translation. The recruitment took place within the authors and through the authors’ network.

**Figure 1:**
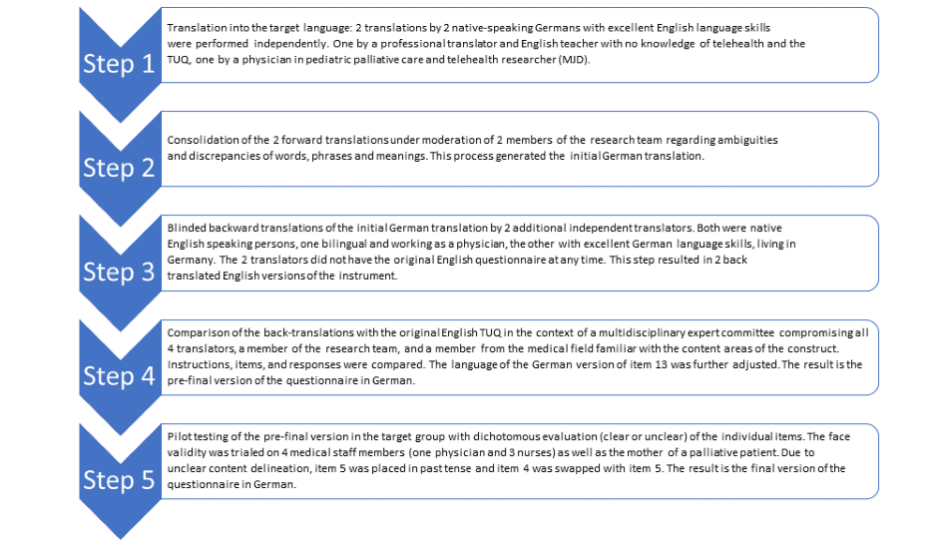
Stage I: the translation process and pilot user testing. TUQ: Telehealth Usability Questionnaire.

To include users’ opinions and views on the German version of the TUQ, we conducted pilot-testing with medical staff, as well as a relative of palliative care children (Figure 1). Several staff members with a high level of clinical experience and patients in palliative care and their relatives were asked to participate in the pilot-testing. The aim of the test was to check whether the items were clear, understandable, and comprehensible. We recruited via the network of authors. The 3 nurses, 1 physician, and 1 relative who participated in the test were asked in an interview to indicate whether the item was clear or not (clear or unclear) and to briefly formulate how they understood the item (thinking aloud method). As a result, we made minor linguistic adjustments.

### Stage II: Development of a Short Scale: Psychometric Testing in Different Target Populations and Final Item Selection

#### Design and Setting

A prospective observational cohort study was conducted on 2 sites—site 1 has an assessment of 13 digital therapeutics, some of which allow direct contact to the physicians of the gastroenterology and rheumatology outpatient clinic at the University Hospital Erlangen. Site 2 has an assessment of the video consultation and auscultation features of a telemedical system with patients and their parents receiving pediatric palliative home care (PPHC) in the German state of Hesse [39,40]. Both sites used modified versions of the TUQ. The survey period was between July 2022 and September 2023.

#### Participants and Patients

All patients who took part in the survey on site 1 were part of 1 of 3 studies conducted by the outpatient clinic at Erlangen University Hospital and were prescribed 1 of the 13 digital therapeutics in the period from January to September 2023 (see Table S1 in Multimedia Appendix 1). All patients who took part in the survey on site 2 were patients with ongoing PPHC. All patients had to first sign the written informed consent. Inclusion criteria for site 1 were a minimum age of 18 years, diagnosis of a rheumatological disease or inflammatory bowel disease, and a prescribed digital therapeutic. The inclusion criteria for site 2 were ongoing PPHC. The exclusion criteria for site 2 were younger than 18 years of age for patients and family members and lack of mental incapacity. Baseline data on demographic characteristics, disease status, and type of digital therapeutics were collected.

#### Data Collection, Procedures, Measurement, and Scales

Baseline data on demographic characteristics, disease status, and usability measured were collected using questionnaires. We calculated the mean and SD for all results. Patients were asked to complete a shortened version of the TUQ before and after the consultation in the ambulance (site 1) or after the use of the telemedical system (site 2). For these studies, 13 TUQ items were selected for their suitability for the general evaluation of telehealth on the basis of expert opinion (see Multimedia Appendix 2 for Cronbach α and Multimedia Appendix 3 for reasons of exclusion). In addition, the single-item NPS [41] was completed on site 1. The survey was completed partly on site or internet-based by email using the REDCap (Research Electronic Data Capture; Vanderbilt University) data collection system (site 1) or the Unipark data collection system (site 2). Participants were asked “How likely are you to recommend this app to other patients?” (original item: “How likely are you to recommend this service?”) and could respond on an 11-point scale ranging from 0 (“Very unlikely”) to 10 (“Very likely”). Depending on the results, patients could be divided into 3 groups—“promoters” (rating 9 or 10), “neutral” (rating 7 or 8), or “detractors” (rating 0 to 6).

The TUQ contains six domains with a total of 21 items, which are thought to represent each individual usability factor (see also Table 1): (1) usefulness (n=3 items), (2) ease of use and learnability (n=3), (3) interface quality (n=4), (4) interaction quality (n=4), (5) reliability (n=3), and (6) satisfaction and future use (n=4).

**Table 1:** English and German versions of the TUQ^a^ for patients^b^.

Item			English		German
**Usefulness (German: Nützlichkeit)**					
	1	Telehealth improves my access to healthcare services.		Die App verbessert meinen Zugang zur Gesundheitsversorgung.	
	2	Telehealth saves me time traveling to a hospital or specialist clinic.		Durch die App spare ich Zeit in ein Krankenhaus oder zu einem niedergelassenen Arzt oder zu einer niedergelassenen Ärztin zu fahren.	
	3	Telehealth provides for my healthcare needs.		Die App kann mich bei meinen gesundheitlichen Anliegen unterstützen.	
**Ease of use and learnability (German: Benutzerfreundlichkeit und Erlernbarkeit)**					
	4	It was simple to use this system.		Die App lässt sich einfach bedienen.	
	5	It was easy to learn to use the system.		Die Bedienung der App war leicht zu erlernen.	
	6	I believe I could become productive quickly using this system.		Ich glaube, ich könnte die App schnell erfolgreich einsetzen.	
**Interface quality (German: Qualität der Benutzeroberfläche)**					
	7	The way I interact with this system is pleasant.		Die Benutzeroberfläche der App ist angenehm gestaltet.	
	8	I like using the system.		Ich bediene die Benutzeroberfläche der App gerne.	
	9	The system is simple and easy to understand.		Die Benutzeroberfläche der App ist einfach und leicht zu verstehen.	
	10	This system is able to do everything I would want it to be able to do.		Die Bedienung der Benutzeroberfläche ermöglicht alles, was ich von ihr erwarte.	
**Interaction quality (German: Qualität der Interaktion)**					
	11	I could easily talk to the clinician using the telehealth system.		Es war einfach, über die App mit dem Gesundheitspersonal zu sprechen.	
	12	I could hear the clinician clearly using the telehealth system.		Über die App konnte ich das Gesundheitspersonal klar und deutlich hören.	
	13	I felt I was able to express myself effectively.		Ich hatte den Eindruck, das Gesundheitspersonal hat mein Anliegen verstanden.	
	14	Using the telehealth system, I could see the clinician as well as if we met in person.		Über die App konnte ich das Gesundheitspersonal genauso gut sehen wie bei einem persönlichen Treffen.	
**Reliability (German: Verlässlichkeit)**					
	15	I think the visits provided over the telehealth system are the same as in-person visits.		Für mich sind Kontakte über die App gleichwertig mit Hausbesuchen.	
	16	Whenever I made a mistake using the system, I could recover easily and quickly.		Wann immer ich einen Fehler bei der Verwendung der App gemacht habe, konnte ich diesen schnell und einfach beheben.	
	17	The system gave error messages that clearly told me how to fix problems.		Die Fehlermeldungen der App sind eindeutig und hilfreich beim Lösen von Problemen.	
**Satisfaction and future use (German: Zufriedenheit und künftige Nutzungsabsicht)**					
	18	I feel comfortable communicating with the clinician using the telehealth system.		Ich fühle mich wohl, wenn ich über die App mit dem Gesundheitspersonal kommuniziere.	
	19	Telehealth is an acceptable way to receive health care services.		Es ist akzeptabel, Gesundheitsversorgung über die App zu erhalten.	
	20	I would use telehealth services again.		Ich würde die App wieder benutzen.	
	21	Overall, I am satisfied with this telehealth system.		Insgesamt bin ich zufrieden mit der App.	

^a^Telehealth Usability Questionnaire.

^b^The German version shown was used for assessing patients receiving PPHC. However, as for the English TUQ, items can be adapted to different health care settings. Pilot-testing resulted in the following change: item 4 was put in the past tense instead of the present tense as shown in the table. Additionally, item 4 was swapped with item 5 (not shown). This version and the translation of the TUQ for health care professionals are free to use following the Creative Commons license 4.0, see Multimedia Appendix 4.

With respect to our initial definition above, the first domain, “usefulness,” measures perceived usefulness in health care. The “efficiency” attribute, proposed by Nielsen [12], aims to assess the “level of attainable productivity of the user after he has learned the system.” We see this attribute covered by TUQ item number 6—“I believe I could become productive quickly using this system” [33,34].

All other attributes are covered in the corresponding domains; however, Nielsen’s [12] “memorability” attribute of usability does not seem to be covered in the TUQ. The TUQ has a Likert scale of 1 “strongly disagree” to 7 “strongly agree” as a response option; there are no reverse-scored items. The development study reports good reliability of the usability factors (usefulness: Cronbach α=0.85; ease of use: Cronbach α=0.93; effectiveness: Cronbach α=0.87; reliability: Cronbach α=0.81; satisfaction: Cronbach α=0.92) [33].

#### Statistical Analysis

Descriptive statistics were performed using SPSS (version 27.0; IBM Corp). EFAs were conducted in R (version 4.3.2; R Core Team [42,43]) using the packages lavaan, semTools, and psych [44,45]. The number of factors was determined using parallel analysis [46]. EFA models were estimated using full information and maximum likelihood information to account for occasional missing values (<5% per case and item). The initial factor solutions were rotated using an oblique Geomin rotation with 100 random starts for the gradient projection algorithm [47]. The Geomin parameter ϵ was set to 0.001, strongly favoring solutions with lower cross-loadings [48-51]. Based on the initial factor solution, a short scale was developed by removing items in order to achieve a simple structure and refine the substantive interpretation of the factors.

### Ethical Considerations

The study was approved by the institutional review board of the Medical Faculty of the University of Erlangen-Nuremberg, Germany (22-425-Bm; January 25, 2023); the University of Kassel, Germany (202213; April 28, 2022); and University of Giessen (AZ 64/22; September 16, 2022). This study is registered in the German Clinical Trials Register (DRKS00030546). Participation in the survey was voluntary. All patients gave their written informed consent before study inclusion. All patients who participated in this study were coded with a consecutive number in a pseudonymization procedure. The data collected were stored and analyzed in a password-protected database. Only previously defined and authorized persons had access to this data. Patients had the option of withdrawing their participation in the study at any time, whereby all personal data were irrevocably deleted. The study was conducted in accordance with the ethical guidelines of the Declaration of Helsinki.

## Results

### Stage I: Translation, Cross-Cultural Adaptation, and Validity

The TUQ was translated in a step-by-step protocol shown in Figure 1. The expert committee discussed several minor cultural and linguistic differences, and the original developer approved all the adjustments. Original TUQ item 4 “It was simple to use this system” was swapped with item 5 “It was easy to learn to use the system,” as multiple pilot user testers were irritated by this pair of questions.

The TUQ questionnaire is designed to assess different types of telehealth and, depending on which technological application is to be assessed, its wording can be adapted accordingly. There was a need to clarify the terms “telehealth,” “system,” and “telehealth system,” which were all replaced by the term “app” to make the questionnaire applicable to apps. See Table 1 for the complete translation.

### Stage II: Development of a Short Scale: Psychometric Testing and Final-Item Selection

#### Patient Characteristics

In total, data from 390 patients were collected in Germany. A total of 41.2% (160/390) were male and the mean age was 41.79 (SD 13.55) years (see Multimedia Appendix 1). All patients used digital therapeutics, except for group 4, which comprised children, adolescents, and young adults with life-limiting illnesses living at home using a telemedical system for video consultation and auscultation [39,40].

#### Descriptive Statistics, Factor Analysis TUQ, and Construction of a TUQ Short Version for Patients (Telehealth Usability and Perceived Usefulness Short Questionnaire for Patients)

Multimedia Appendix 5 provides an overview of the descriptive statistics including the correlations of the items surveyed. Parallel analysis suggested a 2-factor solution, that is, substantially fewer factors than the 6 factors originally proposed for the questionnaire. We also explored 1 and 3-factor solutions but deemed a 2-factor solution most plausible from a substantive point of view. More specifically, the 3-factor solution was defined by a dominant factor that encompassed most of the items, a smaller factor that separated the items of the reliability subscale, and a minor factor that isolated the item “Telehealth saves me time traveling to a hospital or specialist clinic” (see Table 1, item 2). The 1-factor solution blended all aspects together but with 0 loading for item 2. In contrast, the 2-factor solution resulted in 2 equally strong factors. These two factors represented distinct aspects and they are (1) perceived usefulness in health care which refers to the app’s effectiveness in health care, including anticipated future use and (2) usability—pertaining to the user’s experience while operating the app. Both factors explained a substantial proportion of the observed variance (39.8% and 37.2%, respectively) and were positively correlated (*r*=0.59, 95% CI 0.56-0.63). The overall fit of the initial model was good (comparative fit index [CFI]=0.95; standardized root-mean-square residual [SRMR]=0.03; root-mean-square error of approximation [RMSEA]=0.13; *Χ*^2^(53)=385.07; *P*<.001). Further details on the factor loadings and communalities can be found in Multimedia Appendix 6. However, the initial factor loading solution was characterized by many cross-loadings. We removed all items with high cross-loadings from the model in order to sharpen the interpretation, arriving at a short version with 3 items per factor (all considerations are reported in Multimedia Appendix 6). The final version of the scale showed an excellent fit (CFI=0.99; SRMR=0.00; RMSEA=0.02; *Χ*^2^(53)=4.86; *P*=.30). The correlation between the factors was 0.80 (95% CI 0.75-0.85), and the factors explained 41.6% and 40.3% of the total observed variances, respectively. The standardized factor loadings of the final shortened version are displayed in Table 2. The reliability of the factors as estimated by McDonald ω was good for both factors (usability: 0.86 and perceived usefulness in health care 0.89) [52,53]. Cronbach α for the factor’s usability (0.92) and perceived usefulness in health care (0.93) indicates excellent internal consistency.

**Table 2:** Telehealth Usability and Perceived Usefulness Short Questionnaire for Patients (TUUSQ) factor loadings (N=390)^a^.

Item number		Factor loading			Communalities	Attributes
		F1, (95% CI)	F2, (95% CI)			
**Perceived usefulness in health care**						
	Item number 1: The app improves my access to healthcare services.	0.97 (0.89 to 1.04)^b^	–0.1 (–0.19 to –0.02)	0.79		Usefulness
	Item number 2: The app provides for my healthcare needs.	0.91 (0.86 to 0.96)^b^	0.04 (–0.01 to 0.08)	0.88		Usefulness
	Item number 3: I would use the app again.	0.79 (–0.11 to –0.01)^b^	0.14 (0.96 to 1.03)	0.82		Future intention of use
**Usability**						
	Item number 4: It was simple to use the app	–0.06 (–0.07 to 0.23)	0.99 (0.74 to 1.01)^b^	0.91		Ease of use
	Item number 5: The way I interact with the app is pleasant.	0.08 (–0.07 to 0.26)	0.87 (0.58 to 0.89)^b^	0.87		User interface quality
	Item number 6: Whenever I made a mistake using the app, I could recover easily and quickly.	0.09 (0.02 to 0.25)	0.73 (0.68 to 0.90)^b^	0.65		Reliability

^a^English Telehealth Usability Questionnaire (TUQ) items according to the German version referring to “the app.” Factor loadings for the proposed Telehealth Usability and Perceived Usefulness Short Questionnaire for patients (TUUSQ). Factor 1=perceived usefulness in health care and factor 2=usability. The extraction method was oblique Geomin rotation. Adapted from Parmanto et al [33].

^b^Factor loadings above 0.30.

The factors identified in the TUQ show very weak correlations with the NPS—perceived usefulness in health care and NPS—*r*=0.11 (95% CI –0.00 to 0.21); usability with NPS: *r*=–0.11 (95% CI –0.22 to –0.01). Furthermore, the NPS shows no correlation to the majority of the TUQ items and very weak correlations to 6 TUQ items (see Multimedia Appendix 7).

The low strength of these correlations makes it very unlikely that the construct measured by the NPS is similar to the constructs measured by the TUQ (see also Multimedia Appendix 7). To double-check possible relationships between the NPS and the TUQ items, we determined the Net Promoter Score on the basis of the NPS [15] and calculated the Kendall Tau-b coefficients (2-sided). A total of 9 significant negative correlations with small effect size [54] resulted—between items 3 and 11 with a range of *r*=–0.10 to *r*=–0.19.

## Discussion

### Stage I: Complete TUQ Now Available in German in a High-Quality Translation

The complete TUQ was translated into German and cross-culturally adapted. It is comprehensible and equivalent to the English version [33] (see Table 1).

### Stage II: Development of the Telehealth Usability and Perceived Usefulness Short Questionnaire for Patients

We identified 2 factors (“perceived usefulness in health care” and “usability”) in the TUQ which are sufficient to describe the patient’s perception. The NPS does not allow an assessment of usability attributes. The TUQ could be shortened to 6 items without compromising its informativeness as discussed.

### Factor Structure

The TUQ shows a 2-factor structure—on the one hand “usability,” on the other hand “perceived usefulness in health care.” Both factors correlate highly positively (*r*=0.59, 95% CI 0.56-0.63). The same factors were also shown as the main factors of the Spanish version of the TUQ by the working group of Bibiloni et al [36] in 2020. A factor analysis of the Thai version of the TUQ also resulted in a 2-factor model, comparable both to our study and Bibiloni et al [36,55]. The 2 factors were “accessibility” and “utility.” To the best of our knowledge, no further data on the TUQ factor structure were found in other studies [34]. In line with this research, and despite the high correlation of the factors, we deemed the 2-factor solution plausible for 2 reasons—first, it is conceivable that a health care app is technically well-designed but does not fulfill its health-related purpose for the patients. Hence, these aspects are distinguishable, and a 2D questionnaire encourages respondents to think about these aspects separately. In that sense, the high correlation may be an artifact of the context in which patients with highly specialized health care apps were asked to fill in the questionnaire. Second, previous research [56] has shown that factor analysis tends to err in the direction of extracting too few rather than too many factors, thus, the results underline the theoretical notion that there are 2 distinguishable factors in the TUQ.

We removed TUQ items (see Table 1; items 2, 5, 6, 8, 17, 19, and 21) largely due to high cross-loadings in order to sharpen the interpretation, arriving at a short version with 3 items per factor (see Multimedia Appendix 8). As the TUQ, similar to many other usability questionnaires, also contains items measuring the perceived usefulness, we, therefore, propose a clear title stating the dual purpose of the short questionnaire, thus “Telehealth Usability and Perceived Usefulness Short Questionnaire for patients” (TUUSQ). Bibiloni et al [36] proposed a short version with 12 items to assess usability in telehealth focusing on video consultations. This short version shares 4 items with the TUUSQ (see Table 3).

**Table 3:** History of selected TUQ^a^ items^b^.

Item number in TUQ	1	2	3	4	5	7	11	13	14	16	18	19	20	21
Item included in TUUSQ^c^	✓		✓	✓		✓				✓			✓	
Item included by Bibiloni et al [36]	✓	✓		✓	✓		✓	✓	✓	✓	✓	✓	✓	✓
Source of item PSSUQ^d^				✓	✓	✓				✓	✓	✓		✓
Source of item TSQ^e^	✓	✓	✓				✓	✓	✓		✓	✓	✓	✓
Source of item TAM^f^					✓									

^a^TUQ: Telehealth Usability Questionnaire.

^b^The source of the selected TUQ items is shown, as these were originally developed by the authors of the PSSUQ, TSQ, and TAM questionnaires and afterward included in the TUQ [57-59]. TUUSQ items 1, 2, and 3 originate from the TSQ, items 4, 5, and 6 from the PSSUQ questionnaire. Items selected by Bibiloni et al [36] for assessing video consultations also originate from the TAM questionnaire [36].

^c^TUUSQ: Telehealth Usability and Perceived Usefulness Short Questionnaire for patients.

^d^PSSUQ: Post Study System Usability Questionnaire [57].

^e^TSQ: Telemedicine Satisfaction Questionnaire [58].

^f^TAM: Technology Acceptance Model [59].

### Factor Perceived Usefulness in Health Care

The factor that we named “perceived usefulness in health care” was included in the TUUSQ with 2 items relating to health care access and support. In addition, 1 item regarding the intention of future use (Cronbach α=0.79) loads this factor. We interpret this finding as the future intention of use is highly associated with perceived usefulness in health care and not with good usability experience. Other studies in other contexts also demonstrated that satisfaction and future intention of use show a high correlation [60-63].

### Factor Usability

The second factor, “usability,” contains 1 item each regarding ease of use, reliability, and interface quality. The TUUSQ thus lacks the TUQ items addressing the usability attributes as defined by Nielsen [12], that is, “efficiency” (item 6), “efficacy” (item 2), and “satisfaction” (item 21). However, as our data show no benefit in adding further items, and that the feasibility of a 6-item questionnaire is very good; therefore, we advocate this short version. Interestingly, in contrast to the usability attributes, the TUQ item addressing interface quality showed the second-highest factor loading for the factor usability in our study. One of the first usability questionnaires, the “Post Study System Usability Questionnaire” (PSSUQ) showed a 3-factor model with the factors “interface quality,” “system usefulness,” and “information quality” [57]. Saeed et al [64] also showed that, in the context of telehealth home monitoring, the quality of the user interface is of utmost importance for patient usability. Weichbroth [13] reported that among others less commonly assessed usability attributes for the mobile setting include navigation, operability, attractiveness, aesthetics, accessibility, and interaction [13]. Possibly our German translation of the TUQ item 7 “The way I interact with this app is pleasant” which means literally “the app’s user interface has a pleasant design” also relates to these attributes (see Table 1). This finding was also present in usability studies in other health care settings [65].

The original TUQ contains no items regarding memorability. We decided against adding an item to assess memorability as our study design as it does not allow testing for memorability, that is, ease of reusability of the applications after long periods of disuse. Of course, the usefulness of telehealth should be memorable and practical in the longer term, but this question was not part of this study. Further studies on this with a more extended use of telehealth should be carried out in the future.

### NPS in Health Care

Interestingly, the NPS did not show moderate to strong correlations with either the 2 identified factors—perceived usefulness in health care and NPS: 0.105 (95% CI –0.00 to 0.21); usability with NPS: –0.11 (95% CI –0.22 to –0.01) or with any of the individual TUQ items. These results show that the underlying construct of NPS is not associated with any of the patient’s usability or perceived usefulness in health care attributes covered by the TUQ in our studies. As the TUQ contains an item to assess satisfaction (item 21), our studies support the findings of Krol et al [15] that the NPS is not associated with patient satisfaction. The construct that is measured by the NPS in health care continues to remain elusive [66]. Future research assessing health care quality should include the NPS in conjunction with larger surveys [66]. This is necessary since single-item measures are notoriously unreliable—potentially explaining this null finding—and should not be used as a critical variable in high-stakes settings. Moreover, possibly a correlation to other attributes might reveal the underlying construct of the NPS and pave the way to evidence-based use in health care. Finally, it should be noted that Adams et al [66] suggest limiting the use of the NPS to certain health care settings, for example, where patients have a choice of provider. This was not the case in our study.

### Origin and Quality of TUUSQ Items

The items selected from the TUQ for the TUUSQ originate from the PSSUQ and Telehealth Satisfaction Questionnaire (TSQ) questionnaires (see Table 3 [57,58]). All TUUSQ items addressing usability originate from the PSSUQ. The PSSUQ was reviewed by Sousa and Lopez [14] in 2017 and assessed as one of the best available usability questionnaires, although the TUQ was not included in this study [33]. The PSSUQ’s items were generated using an empirical study and showed very good internal consistency (Cronbach α=0.97). However, the quality assessment of validity, reliability, user-centeredness, sample size, and feasibility by Sousa and Rojjansrirat [38] yielded a medium-quality score due to low sample size and lack of reported user-centeredness during item generation. The PSSUQ is also sensitive to user-group and system differences [57].

The TUUSQ items assessing perceived usefulness in health care all originate from the TSQ. The TSQ shows a 3-factor model [58] and all items used by the TUQ and thus TUUSQ belong to the factor called “quality of care provided.” No review of the psychometric properties of the TSQ is available. The reported sample size was low and lacked reported user-centeredness but showed good internal consistency (Cronbach α=0.93).

### Limitations

The TUQ was translated in a way that has proven itself in research. Nevertheless, individual words may seem inappropriate for some target groups (eg, item 15—“Hausbesuche” for “in-person visits”). Depending on the context, “Vor-Ort-Termine,” for example, may seem more appropriate here. We would like to point out that such adjustments can have an impact on the quality of the questionnaire.

The development of the TUUSQ short questionnaire was for the main part only examined patients who received digital therapeutics or used a telehealth system. Further studies are needed to evaluate whether the short questionnaire can be successfully applied more widely in the area of telehealth. Furthermore, the applicability of the TUUSQ may be limited in other cultural contexts, as this study only included patients from Germany. This limitation also applies to the original instruments (PSSUQ and TSQ) used as references. Cultural and contextual differences between the original settings of these instruments and this study could affect their relevance and accuracy in different populations. Thus, we encourage further studies using our translated version to analyze the factor structure in other datasets to investigate the generalizability of our conclusions.

### Conclusions

The TUUSQ offers a short and highly feasible questionnaire for assessing and distinguishing the perceived usefulness and usability of telehealth. The TUUSQ contains solely generalizable items which allow for its use in many different telehealth contexts, as advocated by Sousa and Lopez [14].

Based on our results and the binary factor TUUSQ structure and the recommendations of Sousa and Rojjansrirat [38], we propose the following sequential approach for the assessment of telehealth apps—first, assess whether the app addresses a relevant health care need for patients. If not, and patients report no improved access to health care and health care support, or future intention of use is not reported, the scope of the app should be reevaluated. If perceived usefulness in health care is given, the TUUSQ provides software developers with concrete information on the app’s usability giving feedback on ease of use, reliability, and the quality of the user interface.

Following our study, the TUUSQ can be used in Danish, German, Portuguese, Slovene, Thai, and Urdu, as validated TUQ translations are available for these languages [33,55,67,68]. The TUUSQ is free for commercial and noncommercial use following the Creative Commons license 4.0 [69]. If, besides perceived usefulness in health care and usability, the quality of the video connection during a video consultation, as well as the suitability of this medium are of interest to the researchers, we recommend using the TUQ short version as proposed by Bibiloni et al [36].

## Appendix

Multimedia Appendix 1 Characteristics of participating patients in a prospective observational cohort study at the University Hospital Erlangen and receiving pediatric palliative home care (PPHC) in the German state of Hessen from July 2022 to September 2023 (mean, SD or n, %).

Multimedia Appendix 2 Internal consistency of the Telehealth Usability Questionnaire (TUQ) subscales of the shortened version of the TUQ.

Multimedia Appendix 3 Exclusion reasons for certain Telehealth Usability Questionnaire (TUQ) items. TUQ Items marked with asterisks were included in the shortened version.

Multimedia Appendix 4 German Telehealth Usability Questionnaire version for health care professionals.

Multimedia Appendix 5

Multimedia Appendix 6 Standardized factor loadings and communalities (Comm.) after Geomin rotation for a 2-factor model using the 13 adapted items from the Telehealth Usability Questionnaire (TUQ) for which data were available incl. considerations for removing single items. English TUQ items according to the German version refer to “the app.” N=390. Factor 1= perceived unsefulness in health care and factor 2= usability. The factor correlation for this solution was 0.59. The fit was good overall (SRMR =0.31, CFI =0.95, RMSEA =0.13). Factor loadings above 0.30 are in bold. Adapted from [33]. Items marked with asterisks were included in the short version.

Multimedia Appendix 7 Correlations for NPS and 13 Telehealth Usability Questionnaire (TUQ) items. Pearson correlations. Shortened version of the TUQ [33], see Table S3. NPS: Net Promoter Scale.

Multimedia Appendix 8 German Telehealth Usability and Perceived Usefulness Short Questionnaire version for patients.

## Abbreviations

CFI: comparative fit index
EFA: exploratory factor analysis
ISO: International Organization for Standardization
MARS-G: Mobile App Rating Scale-German
MAUQ: mHealth App Usability Questionnaire
mHealth: mobile health
NPS: Net Promoter Scale
PPHC: pediatric palliative home care
PSSUQ: Post Study System Usability Questionnaire
REDCap: Research Electronic Data Capture
RMSEA: root-mean-square error of approximation
SRMR: standardized root-mean-square residual
SUS: System Usability Scale
TMPQ: Telemedicine Perception Questionnaire
TSQ: Telehealth Satisfaction Questionnaire
TUQ: Telehealth Usability Questionnaire
TUUSQ: Telehealth Usability and Perceived Usefulness Short Questionnaire for patients
