# Supplementary material for: German Version of the Telehealth Usability Questionnaire and Derived Short Questionnaires for Usability and Perceived Usefulness in Health Care Assessment in Telehealth and Digital Therapeutics: Instrument Validation Study
Source: JMIR Hum Factors. 2024 Nov 21;11:e57771. doi: 10.2196/57771 (PMC11621722; doi:10.2196/57771)
Supplement: Multimedia Appendix 1 [file humanfactors_v11i1e57771_app1.docx]

|  | | | | Participating patients *N* = 390 (100%) | | |
| --- | --- | --- | --- | --- | --- | --- |
|  | *n*1 = 128 (33%) | *n*2 = 220 (56%) | *n*3= 30 (8%) | | *n*4 =12 (3%) | Total |
| Gender, female, n (%) | 93 female (73.8%) | 105 female (47.7%) | 21 female (70%) | | 9 female (75%) | 228 female (58.8%) |
| Age in years, mean (SD) | 46.24 ± 11.66 | 39.61 ± 14.29 | 40.83 ±11.09 | | 36.18 ±13.24 | 41.79 ± 13.55 |
| Median [range] | 47 [22; 69] | 56 [18;74] | 40 [25;65] | | 49 [7;56] | 67 [7;74] |
| **Type of Diagnosis, n (%)** |  |  |  | |  |  |
| Rheumatological diseases | 127 (100%) |  | 30 (100%) | |  | 157 (40.3%) |
| IBD (Inflammatory Bowel Disease) |  | Morbus Crohn 127 (58%)  Colitis Ulcerosa 73 (33%) |  | |  | Morbus Crohn 127 (37.8%)  Colitis Ulcerosa 73 (18.8%) |
| Children, adolescents, and young adults with life-limiting illnesses living at home |  |  |  | | 12 (100%) | 12 (3.1%) |
| **Digital therapeutics, n (%)** | 127 (100%) | 220 (100%) | 30 (100%) | | 0 (0%) | 378 (96.9%) |
| MyTargetApp^a^ |  | 220 (100%) |  | |  | 220 (56.6%) |
| ViViRA | 28 (22%) |  | 30 (100%) | |  | 58 (14.9%) |
| Hello Better chronischer Schmerz | 34 (26.8%) |  |  | |  | 34 (8.7%) |
| Somnio | 9 (7.1%) |  |  | |  | 9 (2.3%) |
| NichtRaucher Helden | 10 (7.9%) |  |  | |  | 10 (2.6%) |
| Zanadio | 10 (7.9%) |  |  | |  | 10 (2.6%) |
| Cara Care | 4 (3.1%) |  |  | |  | 4 (1.0%) |
| Deprexis | 4 (3.1%) |  |  | |  | 4 (1.0%) |
| Hello Better Stress | 8 (6.3%) |  |  | |  | 8 (2.1%) |
| Kaia Back Pain | 11 (8.7%) |  |  | |  | 11 (2.8%) |
| Oviva | 8 (6.3%) |  |  | |  | 8 (2.1%) |
| Selfapy | 1 (0.8%) |  |  | |  | 1 (0.3%) |
| Telemedical system for families receiving Pediatric Palliative Home Care |  |  |  | | 12 (100%) | 12 (3.1%) |

^a^ this digital therapeutic was only used by IBD patients.
