# Supplementary material for: German Version of the Telehealth Usability Questionnaire and Derived Short Questionnaires for Usability and Perceived Usefulness in Health Care Assessment in Telehealth and Digital Therapeutics: Instrument Validation Study
Source: JMIR Hum Factors. 2024 Nov 21;11:e57771. doi: 10.2196/57771 (PMC11621722; doi:10.2196/57771)
Supplement: Multimedia Appendix 2 [file humanfactors_v11i1e57771_app2.docx]

| Variable | Cronbach’s Alpha |
| --- | --- |
| Usefulness | .80 |
| Ease of Use and Learnability | .94 |
| Interface Quality | .92 |
| Reliability | .90 |
| Satisfaction and Future Use | .92 |
