# Supplementary material for: German Version of the Telehealth Usability Questionnaire and Derived Short Questionnaires for Usability and Perceived Usefulness in Health Care Assessment in Telehealth and Digital Therapeutics: Instrument Validation Study
Source: JMIR Hum Factors. 2024 Nov 21;11:e57771. doi: 10.2196/57771 (PMC11621722; doi:10.2196/57771)
Supplement: Multimedia Appendix 3 [file humanfactors_v11i1e57771_app3.docx]

| Item |  | Comments |
| --- | --- | --- |
|  | **Usefulness** |  |
| 1. | Telehealth improves my access to healthcare services.* | - |
| 2. | Telehealth saves me time traveling to a hospital or specialist clinic.* | - |
| 3. | Telehealth provides for my healthcare needs.* | - |
|  | **Ease of Use and Learnability** |  |
| 4. | It was simple to use this system.* | - |
| 5. | It was easy to learn to use the system.* | - |
| 6. | I believe I could become productive quickly using this system.* | - |
|  | **Interface Quality** |  |
| 7. | The way I interact with this system is pleasant.* | - |
| 8. | I like using the system.* | - |
| 9. | The system is simple and easy to understand. | Removed after pilot user testing due to redundancy with items 4 and 5 |
| 10. | This system is able to do everything I would want it to be able to do. | Removed due to this item seems inappropriate for the context |
|  | **Interaction Quality** |  |
| 11. | I could easily talk to the clinician using the telehealth system. | Removed due to the application context without video consultation |
| 12. | I could hear the clinician clearly using the telehealth system. | Removed due to the application context without video consultation |
| 13. | I felt I was able to express myself effectively. | Removed due to the application context without video consultation |
| 14. | Using the telehealth system, I could see the clinician as well as if we met in person. | Removed due to the application context without video consultation |
|  | **Reliability** |  |
| 15. | I think the visits provided over the telehealth system are the same as in-person visits. | Removed due to the application context without video consultation |
| 16. | Whenever I made a mistake using the system, I could recover easily and quickly.* | - |
| 17. | The system gave error messages that clearly told me how to fix problems.* | - |
|  | **Satisfaction and Future Use** |  |
| 18. | I feel comfortable communicating with the clinician using the telehealth system. | Removed due to the application context without video consultation |
| 19. | Telehealth is an acceptable way to receive healthcare services.* | - |
| 20. | I would use telehealth services again.* | - |
| 21. | Overall, I am satisfied with this telehealth system.* | - |
