# Supplementary material for: German Version of the Telehealth Usability Questionnaire and Derived Short Questionnaires for Usability and Perceived Usefulness in Health Care Assessment in Telehealth and Digital Therapeutics: Instrument Validation Study
Source: JMIR Hum Factors. 2024 Nov 21;11:e57771. doi: 10.2196/57771 (PMC11621722; doi:10.2196/57771)
Supplement: Multimedia Appendix 4 [file humanfactors_v11i1e57771_app4.docx]

Telehealth Usability Questionnaire for healthcare professionals

German version

**Inwieweit treffen die folgenden Aussagen auf Ihre Erfahrungen zu? Kreuzen Sie an!**

Zur Beurteilung haben Sie jeweils folgende Skala zur Verfügung:

1  2  3  4  5  6  7

stimme überhaupt stimme voll zu

nicht zu

**Es gibt weder "richtige" noch "falsche" Antworten.**

1. Die App verbessert meinen Zugang zur Patientenversorgung.

1  2  3  4  5  6  7

stimme überhaupt stimme voll zu

nicht zu

1. Durch die App spare ich Zeit zu einem Patienten zu fahren.

1  2  3  4  5  6  7

stimme überhaupt stimme voll zu

nicht zu

1. Die App kann bei gesundheitlichen Anliegen unterstützen.

1  2  3  4  5  6  7

stimme überhaupt stimme voll zu

nicht zu

1. Die Bedienung der App war leicht zu erlernen.

1  2  3  4  5  6  7

stimme überhaupt stimme voll zu

nicht zu

1. Die App lässt sich einfach bedienen.

1  2  3  4  5  6  7

stimme überhaupt stimme voll zu

nicht zu

1. Ich glaube, ich könnte die App schnell erfolgreich einsetzen.

1  2  3  4  5  6  7

stimme überhaupt stimme voll zu

nicht zu

1. Die Benutzeroberfläche der App ist angenehm gestaltet.

1  2  3  4  5  6  7

stimme überhaupt stimme voll zu

nicht zu

1. Ich bediene die Benutzeroberfläche der App gerne.

1  2  3  4  5  6  7

stimme überhaupt stimme voll zu

nicht zu

1. Die Benutzeroberfläche der App ist einfach und leicht zu verstehen.

1  2  3  4  5  6  7

stimme überhaupt stimme voll zu

nicht zu

1. Die Bedienung der Benutzeroberfläche ermöglicht alles, was ich von ihr erwarte.

1  2  3  4  5  6  7

stimme überhaupt stimme voll zu

nicht zu

1. Es war einfach, über die App mit dem Patienten zu sprechen.

1  2  3  4  5  6  7

stimme überhaupt stimme voll zu

nicht zu

1. Über die App konnte ich den Patienten klar und deutlich hören.

1  2  3  4  5  6  7

stimme überhaupt stimme voll zu

nicht zu

1. Ich hatte den Eindruck, mich über die App gut ausdrücken zu können.

1  2  3  4  5  6  7

stimme überhaupt stimme voll zu

nicht zu

1. Über die App konnte ich den Patienten genauso gut sehen wie bei

einem persönlichen Treffen.

1  2  3  4  5  6  7

stimme überhaupt stimme voll zu

nicht zu

1. Für mich sind Kontakte über die App gleichwertig mit Hausbesuchen.

1  2  3  4  5  6  7

stimme überhaupt stimme voll zu

nicht zu

1. Wann immer ich einen Fehler bei der Verwendung der App gemacht habe,

konnte ich diesen schnell und einfach beheben.

1  2  3  4  5  6  7

stimme überhaupt stimme voll zu

nicht zu

1. Die Fehlermeldungen der App sind eindeutig und hilfreich beim Lösen von Problemen.

1  2  3  4  5  6  7

stimme überhaupt stimme voll zu keine Angabe

nicht zu möglich

1. Ich fühle mich wohl, wenn ich über die App mit dem Patienten kommuniziere.

1  2  3  4  5  6  7

stimme überhaupt stimme voll zu

nicht zu

1. Es ist akzeptabel, Gesundheitsversorgung über die App zu leisten.

1  2  3  4  5  6  7

stimme überhaupt stimme voll zu

nicht zu

1. Ich würde die App wieder benutzen.

1  2  3  4  5  6  7

stimme überhaupt stimme voll zu

nicht zu

1. Insgesamt bin ich zufrieden mit der App.

1  2  3  4  5  6  7

stimme überhaupt stimme voll zu

nicht zu
