# Supplementary material for: German Version of the Telehealth Usability Questionnaire and Derived Short Questionnaires for Usability and Perceived Usefulness in Health Care Assessment in Telehealth and Digital Therapeutics: Instrument Validation Study
Source: JMIR Hum Factors. 2024 Nov 21;11:e57771. doi: 10.2196/57771 (PMC11621722; doi:10.2196/57771)
Supplement: Multimedia Appendix 5 [file humanfactors_v11i1e57771_app5.docx]

| 14 | 378 | 6.63 | 2.97 | 0.12* | 0.17** | 0.07 | -0.11* | -.011* | 0.03 | -0.11* | 0.05 | -0.05 | -0.06 | -0.05 | 0.13* | 0.08 | - |
| --- | --- | --- | --- | --- | --- | --- | --- | --- | --- | --- | --- | --- | --- | --- | --- | --- | --- |
| 13 | 390 | 3.06 | 2.00 | 0.77** | 0.33** | 0.84** | 0.81** | 0.82** | 0.84** | 0.84** | 0.87** | 0.71** | 0.64** | 0.76** | 0.90** | - |  |
| 12 | 390 | 2.98 | 2.09 | 0.79** | 0.42** | 0.84** | 0.71** | 0.72** | 0.81** | 0.74** | 0.82** | 0.65** | 0.59** | 0.72** | - |  |  |
| 11 | 390 | 3.21 | 1.85 | 0.68** | 0.30** | 0.75** | 0.71** | 0.72** | 0.74** | 0.73** | 0.72** | 0.65** | 0.67** | - |  |  |  |
| 10 | 390 | 3.44 | 1.71 | 0.55** | 0.25** | 0.59** | 0.67** | 0.68** | 0.67** | 0.68** | 0.67** | 0.80** | - |  |  |  |  |
| 9 | 390 | 3.30 | 1.94 | 0.56** | 0.16** | 0.63** | 0.76** | 0.76** | 0.72** | 0.75** | 0.75** | - |  |  |  |  |  |
| 8 | 390 | 3.23 | 1.94 | 0.74** | 0.29** | 0.79** | 0.85** | 0.85** | 0.84** | 0.87** | - |  |  |  |  |  |  |
| 7 | 390 | 3.21 | 2.23 | 0.66** | 0.13* | 0.73** | 0.89** | 0.88** | 0.80** | - |  |  |  |  |  |  |  |
| 6 | 390 | 2.98 | 2.02 | 0.75** | 0.32** | 0.81** | 0.79** | 0.80** | - |  |  |  |  |  |  |  |  |
| 5 | 390 | 3.14 | 2.41 | 0.64** | 0.12* | 0.72** | 0.97** | - |  |  |  |  |  |  |  |  |  |
| 4. | 390 | 3.18 | 2.43 | 0.61** | 0.09 | 0.71** | - |  |  |  |  |  |  |  |  |  |  |
| 3 | 390 | 2.88 | 1.80 | 0.83** | 0.38** | - |  |  |  |  |  |  |  |  |  |  |  |
| 2 | 390 | 2.89 | 1.77 | 0.53** | - |  |  |  |  |  |  |  |  |  |  |  |  |
| 1 | 390 | 2.82 | 1.71 | - |  | 1 | 390 | 2.82 | 1.71 | - |  | 1 | 390 | 2.82 | 1.71 | - |  |
| Variable | *n* | *M* | *SD* | 1 TUQ 1 | 2 TUQ 2 | 3 TUQ 3 | 4 TUQ 4 | 5 TUQ 5 | 6 TUQ 6 | 7 TUQ 7 | 8 TUQ 8 | 9 TUQ 16 | 10 TUQ 17 | 11 TUQ 19 | 12 TUQ 20 | 13 TUQ 21 | 14 NPS |
