# Supplementary material for: German Version of the Telehealth Usability Questionnaire and Derived Short Questionnaires for Usability and Perceived Usefulness in Health Care Assessment in Telehealth and Digital Therapeutics: Instrument Validation Study
Source: JMIR Hum Factors. 2024 Nov 21;11:e57771. doi: 10.2196/57771 (PMC11621722; doi:10.2196/57771)
Supplement: Multimedia Appendix 6 [file humanfactors_v11i1e57771_app6.docx]

| Item | | Factor loading | | Comm. | Comments |
| --- | --- | --- | --- | --- | --- |
|  | | Factor 1 [95 %CI] | Factor 2 [95% CI] |  |  |
| Item no. 1*: The app improves my access to healthcare services. | | **0.79**  **[0.74; 0.84]** | 0.13  [0.07; 0.18] | 0.76 | – |
| Item no. 2: The app saves me time traveling to a hospital or specialist clinic. | **0.75**  **[0.68; 0.83]** | | **-0.38**  **[-0.47; -0.29]** | 0.37 | Removed due to high cross-loading and low communality |
| Item no. 3*: The app provides for my healthcare needs. | | **0.73**  **[0.68; 0.77]** | 0.26  [0.21; 0.32] | 0.82 | – |
| Item no. 4*: It was simple to use the app. | | 0.06  [0.02; 0.09] | **0.95**  **[0.93; 0.98]** | 0.97 | – |
| Item no. 5: It was easy to learn to use the app. | | 0.09  [0.06; 0.13] | **0.92**  **[0.90; 0.95]** | 0.96 | Removed to shorten the questionnaire |
| Item no. 6: I believe I could become productive quickly using the app. | | **0.56**  **[0.50; 0.61]** | **0.46**  **[0.40; 0.51]** | 0.81 | Removed due to high cross-loading |
| Item no. 7*: The way I interact with the app is pleasant. | | 0.28  [0.22; 0.32] | **0.74**  **[0.69; 0.78]** | 0.85 | – |
| Item no. 8: I like using the app. | | **0.47**  **[0.42; 0.52]** | **0.57**  **[0.52; 0.62]** | 0.86 | Removed due to high cross-loading |
| Item no. 9*: Whenever I made a mistake using the app, I could recover easily and quickly. | | 0.28  [0.20; 0.35] | **0.61**  **[0.54; 0.67]** | 0.65 | Preferred over item 5 because it covers error handling as a unique aspect of usability that is often covered by definitions of usability |
| Item no. 10: The app gave error messages that clearly told me how to fix problems. | | **0.32**  **[0.23; 0.40]** | **0.50**  **[0.42; 0.58]** | 0.524 | Removed due to high cross-loading |
| Item no. 11: The app is an acceptable way to receive healthcare services. | | **0.51**  **[0.44; 0.58]** | **0.40**  **[0.33; 0.47]** | 0.66 | Removed due to high cross-loading |
| Item no. 12*: I would use the app again. | | **0.78**  **[0.73; 0.82]** | 0.23  [0.18; 0.29] | 0.87 | – |
| Item no. 13: Overall, I am satisfied with the app. | | **0.62**  **[0.58; 0.67]** | **0.44**  **[0.39; 0.48]** | 0.89 | Removed due to high cross-loading |
