# Supplementary material for: German Version of the Telehealth Usability Questionnaire and Derived Short Questionnaires for Usability and Perceived Usefulness in Health Care Assessment in Telehealth and Digital Therapeutics: Instrument Validation Study
Source: JMIR Hum Factors. 2024 Nov 21;11:e57771. doi: 10.2196/57771 (PMC11621722; doi:10.2196/57771)
Supplement: Multimedia Appendix 7 [file humanfactors_v11i1e57771_app7.docx]

| Item | NPS |  |
| --- | --- | --- |
|  | [95% CI] | *P* value |
| Item no. 1: The app improves my access to healthcare services. | 0.11  [0.01; 0.21] | .04 |
| Item no. 2: The app saves me time traveling to a hospital or specialist clinic. | 0.16  [0.06; 0.26] | <.001 |
| Item no. 3: The app provides for my healthcare needs. | 0.06  [-0.05; 0.16] | .28 |
| Item no. 4: It was simple to use the app. | -0.12  [-0.22; -0.02] | .02 |
| Item no. 5: It was easy to learn to use the app. | -0.12  [-0.22; -0.02] | .02 |
| Item no. 6: I believe I could become productive quickly using the app. | 0.02  [-0.08; 0.12] | .68 |
| Item no. 7: The way I interact with this app is pleasant. | -0.10  [-0.20; 0.00] | .05 |
| Item no. 8: I like using the app. | 0.03  [-0.07; 0.13] | .53 |
| Item no. 9: Whenever I made a mistake using the app, I could recover easily and quickly. | -0.06  [-0.16; 0.04] | .26 |
| Item no. 10: The app gave error messages that clearly told me how to fix problems. | -0.06  [-0.16; 0.04] | .23 |
| Item no. 11: The app is an acceptable way to receive healthcare services. | -0.05  [-0.15; 0.06] | .37 |
| Item no. 12: I would use the app again. | 0.12  [0.02; 0.22] | .02 |
| Item no. 13: Overall, I am satisfied with the app. | 0.08  [-0.03; 0.18] | .15 |
