# Supplementary figures and images for: German Version of the Telehealth Usability Questionnaire and Derived Short Questionnaires for Usability and Perceived Usefulness in Health Care Assessment in Telehealth and Digital Therapeutics: Instrument Validation Study
Source: JMIR Hum Factors. 2024 Nov 21;11:e57771. doi: 10.2196/57771 (PMC11621722; doi:10.2196/57771)

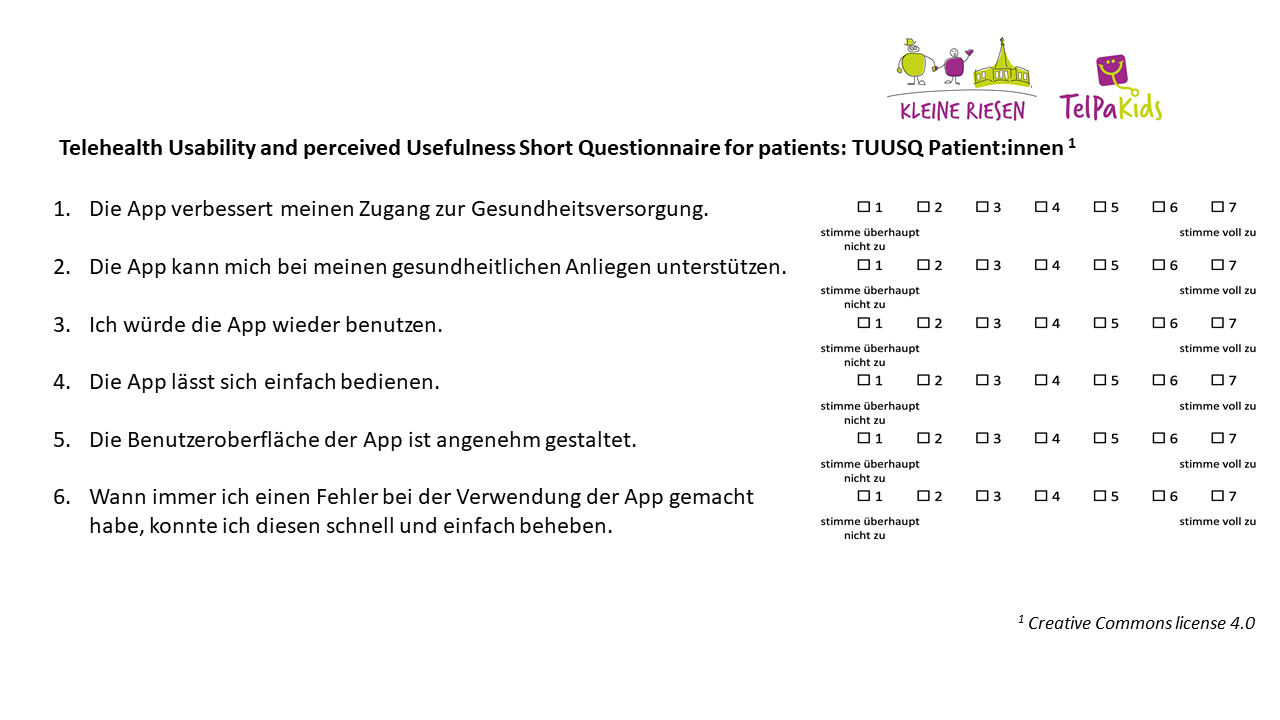

Supplement: Multimedia Appendix 8 [file humanfactors_v11i1e57771_app8.docx]
